# Trust or Distrust: The Effect of Facial Emotion and Trustworthy Behavior on Trust Decision-Making

**DOI:** 10.5334/pb.1214

**Published:** 2023-08-17

**Authors:** Mengmeng Zhou, Yixin Hu, Dawei Wang

**Affiliations:** 1 School of Psychology, Shandong Normal University, Jinan, CN

**Keywords:** trust game, facial emotions, trustworthy behavior, emotional arousal, trust decision making

## Abstract

Based on the model of emotion as social information, this study explores the effects of facial emotions and trustworthy behavior on trust decision-making in trust game through two experiments. The present study used trust game explores the impact of players’ facial emotion, arousal and trustworthy behavior on individual trust decision-making through two experiments. The results can be summed up as follows: (1) in the repeated interaction with four players, individuals invest more in trustworthy players than untrustworthy players; (2) individuals invested more in trustworthy players with happy facial emotions, while untrustworthy players with angry facial emotions received less investment. High-arousal facial emotion results in a more extreme investment by the individual; (3) when the players’ facial emotion and behavior are inconsistent, the individual will judge according to the player’s current behavior, rather than the facial emotion or past behavior.

Trust decision-making is the process by which individuals make decisions based on the information received by people when they interact with others. The available research on trust decision-making involves a single interaction with different partners (Fu et al., 2018; van ’t Wout & Sanfey, 2008) and repeated interactions with the same person (Campellone & Kring, 2013; Chang et al., 2010; Engle-Warnick & Slonim, 2004; King-Casas et al., 2005; Wang et al., 2015). However, in real life, most social activities experienced by individuals involve a series of repeated interactions. In repeated interactions, individuals can constantly obtain information about each other, which provides updated evidence for individuals to make choices (Keltner & Kring, 1998). In addition, a previous study indicated that the amount of information received influences trust decisions or the subsequent behavior of individuals (Schaffer et al., 2018). The trust game is a classic paradigm used to research trust decision-making and cooperative behavior and psychology (Alós-Ferrer & Farolfi, 2019; Berg et al., 1995; Johnson & Mislin, 2011), which is especially suitable for exploring how people make trust choices in their interactions with partners. This game involves two players, A and B. Player A is endowed with an initial amount of money, $10, and can choose to invest any amount of endowment to B. The amount that player A is investing in B will be multiplied by a factor, usually 3 or 4, and then player B can choose the amount that they will return to Player A. In this game, trust is operationally defined as the amount invested by player A, and trustworthiness is represented as the amount returned to player A. A previous study showed that among the 32 participants in the role of player A, 30 chose to invest some amount of money in player B, and the average investment by player A was $5.16 (Campellone & Kring, 2013). Therefore, while they have no information about each other, most of the participants in the role of player A chose to invest a part of the endowment in player B, hoping that the player will reciprocate the trust. Nevertheless, whether the behavior of player B is trustworthy is still an important factor in determining the investment amount by player A in a trust game. Hence, we proposed our hypothesis that:

H1: Participants will invest significantly more in trustworthy players than in untrustworthy players.

The model of emotion as social information points out that just as emotion provides information for decision-making (Schwarz & Clore, 1983), facial emotion can provide implicit information for an individual, which may, in turn, affect their behavior (Kleef & Gerben, 2009). Empirical work on facial expressions and trust decision-making provided further evidence (Campellone & Kring, 2013; Chang et al., 2010). Researchers found that the credibility judgements based on facial features are made rapidly (Todorov et al., 2009), are reliable (Brownlow, 1992), and are thought to play a vital role in survival (Cosmides & Toobey, 2000). Relevant research has demonstrated that facial features can convey information and social intentions of partners, such as personality characteristics and complex social characteristics, in repeated social interactions (Frith & Frith, 1999). Existing research shows that in a trust game, participants are more inclined to pay some amount of money in exchange for photos of their partners (Eckel & Petrie, 2011; Ewing et al., 2015). In a social interaction, facial emotions can convey information that may affect trust decisions (Keltner & Haidt, 1999; Pegna et al., 2019), suggesting that individuals believe that facial emotions can improve the accuracy of trust decision (Carragher et al., 2017). There had been some research on the effect of facial emotions on trust decision-making, which were conducted in Western culture. For example, researchers found that social partners with a smiling facial expression experience more cooperation and trustworthy behavior (Scharlemann et al., 2001). Angry facial expressions keep individuals away from them (Marsh et al., 2005). People generally think that individuals with angry facial emotions are untrustworthy (Dunn & Schweitzer, 2005) and rarely show approaching behavior (Montepare & Dobish, 2003). However, Li et al. (2022) discovered that in Chinese culture, people invested more money in happy faces compared to sad ones. The model of “Feeling as information” also tends to believe that the valence of emotion as information plays a role in the individual decision-making process (Clore et al., 2001; Schwarz & Clore, 1983). In summary, we proposed our hypothesis that:

H2: The facial emotion of players will affect the investment amount of individuals. Happy facial emotion will receive more investment amount, while angry facial emotion will receive less investment amount.

Indeed, facial displays of emotion convey information that can influence behavior (Keltner & Haidt, 1999; Pegna et al., 2019). Moreover, existing studies have indicated that the amount of reciprocation influences trust decisions (Campellone & Kring, 2013; Chang et al., 2010; King-casas et al., 2005). In social encounters, however, it is impossible for people to process only behavioral information in their interaction with a partner. Studies have indicated that the amount of information received influences trust decisions or the subsequent behavior of individuals (Schaffer et al., 2018; Wilson & Eckel, 2023). Therefore, how facial emotion and trustworthy behavior jointly influence an individual’s trust decision-making is worthwhile to explore. When the facial emotional expression and trustworthy behavior are concordant, for example, when the partner is trustworthy with happy facial emotion (or trustworthy facial features), people are more incline to invest money in their social partner (Campellone &Kring, 2013; Chang et al. 2010). However, when the facial emotional information and behavior of the partner are inconsistent, do individuals pay more attention to facial emotional information or behavioral information? Existing research shows that when facial emotions (or facial features) and behaviors are in conflict, individuals pay more attention to the behavioral information of their partners (Campellone & Kring, 2013; Chang et al., 2010). In addition, previous studies showed that initial judgement (including facial expressions) may influence the way information from repeated interactions is updated (Delgado et al., 2005). Except for the facial expression of emotions and ongoing behavior, other social characteristics also influence trust choices. For example, knowing that a social partner has engaged in immoral behaviors in the past, regardless of trustworthy behavior, individuals will place less trust in a partner in the iterated trust game (Delgado et al., 2005). In addition, how do people make decisions when the interacting partner’s trustworthy behavior is inconsistent between previous and current interactions? Indeed, most social interaction scenarios involve an ongoing series of exchanges that provide new information that, in turn, shapes decisions (Keltner & Kring, 1998). When deciding whether to trust a person, research suggests that we continue to modify our decision-making throughout repeated interactions, updating the information of social partners’ trustworthy behavior (King-casas, et al., 2005). Therefore, the answer to how people make decision when previous and current behaviors are incongruent is that people make decision according to the ongoing behavior of the partner. Hence, we proposed our hypothesis that:

H3: When facial emotion and behavioral information are incongruent, participants are more inclined to rely on behavioral information to make trust choices.

Existing research on the facial display of emotion and trust decision-making mainly focus on the emotional valence. According to the dimension theory of emotion (Russell, 1980), arousal, as the other dimension of emotion, will also have an impact on high-level perception, for example, time perception (Gan, Luo, & Zhang, 2009; Stefanucci & Storbeck, 2009). Reported findings showed that, as an important dimension of emotional representation, arousal might also participate in the individual decision-making process (Wang et al., 2013). At present, the ways by which emotional arousal can be manipulated include picture stimulation and the size of the task (Wang et al., 2013). Risk decision-making mainly takes the gambling amount as emotional arousal (FeldmanHall et al., 2016; Huang et al., 2018; Wang et al., 2013). Existing studies have shows that the decision-making preference is regulated by the level of emotional arousal. Individuals prefer ambiguity when facing risk and ambiguity decision-making under high emotional arousal (Wang et al., 2013), which will increase risk-taking behavior (FeldmanHall et al., 2016). At the same time, work on uncertain decision-making showed that when the decision-making is high-risk, increasing arousal can reduce risky behavior (FeldmanHall et al., 2016; Huang et al., 2018). The above research showed that emotional arousal has an impact on risky decisions. Moreover, trust decisions often endure considerable risk and uncertainty (Chang et al., 2010; Eckel & Wilson, 2004). Therefore, we speculated that emotional arousal would have an impact on trust decisions.

H4: The arousal of facial emotion influences participants’ trust choices. Players with high-arousal happy facial emotion will receive more investment than those with low-arousal happy facial emotion.

In addition, recent evidence suggests that women are more risk-averse than men in social risk-taking decisions (Friedl et al., 2019). Particularly in domains such as economics (Figner & Weber, 2011), in contrast to men, women may be less inclined to trust others with financial decision-making. A recent meta-analysis study showed that men invested more money in the trust game than women (Van Den Akker et al., 2020). Additionally, research on the Investment Game found that men were more likely to trust others than women (Buchan, Croson & Solnick, 2008). Therefore, we assume that gender plays a role in trust decisions.

H5: Women will invest less in players than men.

To date, most studies have focused on manipulating the facial emotions or trustworthiness of social partners, and few have considered the roles of both in trust decision-making. However, in order to illustrate the factors affecting trust decisions, we need to understand how facial emotion and trustworthy behavior affect trust decisions. When facial emotions and trustworthy behavior are explored simultaneously, the amount of money an individual invests changes when these two factors are congruent (e.g., a player with happy facial emotion and trustworthy behavior) or conflicting (e.g., a player with angry facial emotion and untrustworthy behavior). This helps us to understand how facial emotion and trustworthy behavior shape repeated trust decision-making.

## The Present Study

The present study examined the above questions via two experiments adopting a trust game that was divided into two versions, facial emotion first (the first block of this version was present in the facial emotion of players; FF) and behavior first (the first block of this version was absent in the facial emotion of players; BF). Experiment 1 verified the effect of happy and angry facial emotions and trustworthy behavior on trust decision-making in a Chinese cultural background. Designed on the basis of experiment 1 and adding an arousal variable, experiment 2 explored the influence of the high and low arousal of happy and angry emotional displays and trustworthy behavior on trust decisions.

## Experiment 1 The Effect of Happy and Angry Facial Emotion and Trustworthy Behavior on Trust Decision-Making

### Methods

#### Participants

We conducted a power analysis using G*power following prior studies (Campellone & Kring, 2013; Faul et al., 2007), with the result showing that 1–β = 0.99 (effect size = 0.25, alpha = 0.05, total sample size = 59, number of groups = 2, number of measurements = 4, corr among rep measures = 0.5, nonsphericity correction ɛ = 1). Fifty-nine undergraduates (*M*_age_ = 19.82, *SD*_age_ = 0.87, 30 males) from Shandong Normal University participated in the present study and received gifts for their participation in the experiment.

All human participating procedures used in the present study were conducted in the light of the ethical standards of the academic committee of Shandong Normal University and certify that the study was performed in accordance with the ethical standards as laid down in the 1964 Declaration of Helsinki and its later amendments or comparable ethical standards. Participants written the informed consent and were informed that they can terminate the experiment at any time.

#### Experimental Design

A 2 (facial emotion: happy vs angry) × 2 (partner’s behavior: trustworthy vs untrustworthy) × 2 (version: behavior first vs facial emotion first) mixed design was adopted in the current study with version as a between-subject variable and facial emotion and partner’s behavior as within-subject variables. The dependent variable is the amount of the participant’s investment.

The pictures in the current study were selected from the Chinese emotional picture system revised by Bai et al. (2005). Two happy and two angry facial emotion pictures were selected, half were men and half were women, which were consistent in arousal and attractiveness (two with happy facial emotion; arousal was 6.67 and attractiveness was 6.84 and 5.51, respectively; two with angry facial emotion; arousal was 7.33 and 8.33, respectively, and attractiveness was 4.11 and 4.50, respectively. See Appendix A).

The trust game adopted by Campellone and Kring (2013), which was divided into two versions: BF and FF, was employed in the present study. The two key principles to distinguish the BF and FF versions are whether a player’s facial emotion was presented prior to trustworthy behavior and whether there was consistency between facial emotion and trustworthy behavior.

##### Behavior first version

In the first block of the BF version, participants interacted with four players with no facial emotion. In this block two out of four were trustworthy (the reciprocate is random from one to two times the participant’s investment), and the other two were untrustworthy (the reciprocate is random from zero to a half that of the participant’s investment). In the second block, participants interacted with the same four players in the first block, in which players’ trustworthiness were same as the first block, but facial emotion (happy or angry) was presented.

##### Facial emotion first version

In the first block of the FF version, participants interacted with four players with facial emotion. The facial emotion and trustworthiness of the players were congruent, that is, players with the facial display of happiness were trustworthy, and players with the angry facial display were untrustworthy. In the second block, participants interacted with the same four players in the first block, and only presented the number of players without facial displays of emotion. In addition, two players went from being trustworthy in the first block to untrustworthy in the second block, while the other two were opposite. See more details in Table 1.

**Table 1:** Facial emotion and trustworthiness of four players in two version.


PLAYER	BEHAVIOR FIRST		FACIAL EMOTION FIRST	
	
	BLOCK 1	BLOCK 2	BLOCK 1	BLOCK 2

Player 1	TR	TR+HAP	TR+HAP	TR

Player 2	UN	UN+HAP*	UN+ANG	TR*

Player 3	TR	TR+ANG*	TR+HAP	UN*

Player 4	UN	UN+ANG	UN+ANG	UN


*Notes:* TR = trustworthy, UN = untrustworthy; HAP = happy facial emotion; ANG = angry facial emotion; * = inconsistent.

#### Procedure

The task materials were presented on a Dell laptop computer, and the experimental process was controlled by E-Prime 2.0.

**Figure 1:**
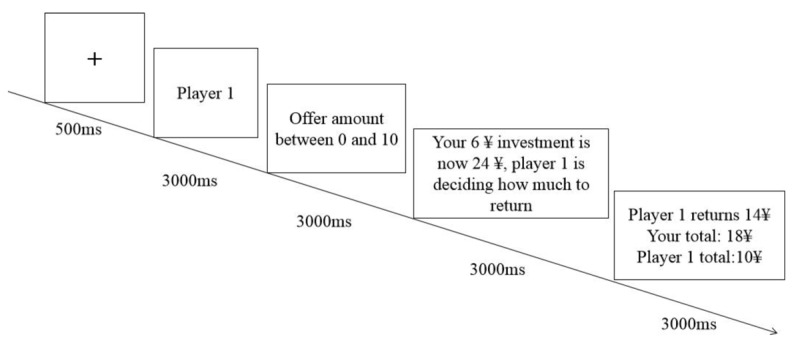
Example of experiment trial.

After providing informed consent, the participants were informed that they would play computer games with others. The participants were guided to believe that the other players were real people. Each player was simulated and only acted according to a predetermined pattern. The participants were told that they would see randomly selected pictures of other players in part of the trials. There were two programs in the experiment, each program contained two blocks, and each block contained four players. The participants had to carry out 16 trials with each player, and each block had 64 trials for a total of 128 trials. See Figure 1.

#### Data Analysis

Statistical analyzes were performed using SPSS 16.0.

### Results

According to the first hypothesis, participants will invest significantly more in trustworthy players than in untrustworthy players. The current study analyzed the first block of the BF version, and the results showed that the amount invested in trustworthy players (7.56 ± 2.67) was significantly greater than that in untrustworthy players (3.24 ± 2.84), *t* (881) = 29.51, *p* < 0.001, *d* = 1.57.

To test the second hypothesis, the present study analyzed the first blocks of FF version and BF version via repeated ANOVA of the 4 (player) × 2 (version) × 2 (gender) design. The results showed that the main effect of player was significant, *F* (3, 881) = 1212.81, *p* < 0.001, *η_p_^2^* = 0.58, the main effects of gender and version were not significant. The interaction between player and version was significant, *F*(3, 881) = 33.74, *p* < 0.001, *η_p_^2^* = 0.037. Further simple effect analysis showed that for player 1 and player 3, the amount of investment in the FF version (8.10; 8.31) was significantly higher than that in BF version (7.56; 7.51); for player 2 and player 4, the amount of investment in the FF version (2.17; 2.24) was significantly lower than that in the BF version (3.21; 3.26). The interaction between player and gender was significant, *F*(3, 881) = 16.07, *p* < 0.001, *η_p_^2^* = 0.018. Further simple effect analysis showed that for player 1 and player 3, the investment amount by male participants (8.14; 8.28) was higher than that invested by female participants (7.51; 7.54), while for player 2 and player 4, the investment amount by male participants (2.46; 2.49) was significantly less than that invested by female participants (2.92; 3.01). The interaction between gender and version was significant, *F*(3, 881) = 4.59, *p* < 0.05, *η_p_^2^ = 0.005*. Further simple effect analysis showed that in the BF version, the investment amount by male participants (5.53) was significantly higher than that invested by female participants (5.24), and there was no significant difference in the FF version. See Table 2 for details.

**Table 2:** The amount of investment under different conditions in the two versions of block1 (*M* ± *SD*).


GENDER	VERSION	PLAYER 1(TR)	PLAYER 2(UN)	PLAYER 3(TR)	PLAYER 4(UN)

male	Behavior	8.04 ± 2.50	2.91 ± 2.78	8.04 ± 2.22	3.14 ± 3.06

	Facial emotion	8.25 ± 2.47	2.01 ± 2.85	8.52 ± 2.17	1.84 ± 2.44

female	Behavior	7.07 ± 2.80	3.51 ± 2.63	6.97 ± 2.95	3.39 ± 2.84

	Facial emotion	7.95 ± 2.46	2.33 ± 2.41	8.10 ± 2.17	2.63 ± 2.49


*Notes:* TR = trustworthy, UN = untrustworthy.

To test the third hypothesis, we separately analyzed the BF version and FF version. First, we carried out a repeated ANOVA of the 4 (player) × 2 (block) × 2 (gender) design for the BF version. The results showed that the main effect of player was significant, *F*(3, 382) = 1014.479, *p* < 0.001, *η_p_^2^* = 0.73, and the main effect of block was significant, *F*(1, 382) = 57.04, *p* < 0.001, *η_p_^2^* = 0.13. The main effect of gender was significant, *F*(1, 382) = 21.32, *p* < 0.001, *η_p_^2^* = 0.053. The interaction between block and player was significant, *F*(3, 382) = 70.44, *p* < 0.001, *η_p_^2^* = 0.16. Further simple effect analysis showed that the amount of investment in player 1 (7.54, 7.95) and player 3 (7.50, 8.01) was significantly larger than that in player 2 (3.19, 1.55) and player 4 (3.26, 1.54) in both block 1 and block 2. The interaction between player and gender was significant, *F*(3, 382) = 30.86, *p* < 0.001, *η_p_^2^* = 0.075. Further simple effect analysis showed that for player 1 and player 3, the investment amount by male participants (8.43, 8.46) was significantly higher than invested by female participants (7.06, 7.05), while for player 2 and player 4, the investment amount by female participants (2.61, 2.65) was significantly higher than that invested by male participants (2.13, 2.16). See Table 3 for details.

**Table 3:** The amount of investment under different conditions in BF versions (*M* ± *SD*).


GENDER	BLOCK	PLAYER 1(TR)	PLAYER 2(UN*)	PLAYER 3(TR*)	PLAYER 4(UN)

male	1	8.11 ± 2.38	2.91 ± 2.73	8.12 ± 2.16	3.09 ± 3.04

	2	8.76 ± 1.91	1.34 ± 1.92	8.79 ± 2.01	1.24 ± 1.97

female	1	6.98 ± 2.82	3.46 ± 2.48	6.87 ± 2.95	3.44 ± 2.78

	2	7.15 ± 2.48	1.76 ± 2.06	7.23 ± 2.20	1.85 ± 2.23


*Notes:* TR = trustworthy, UN = untrustworthy; * = inconsistent.

In addition, we also performed a repeated ANOVA of the 4 (player) × 2 (block) × 2 (gender) design for FF version. The results showed that the main effect of player was significant, *F*(3, 401) = 930.78, *p* < 0.001, *η_p_^2^* = 0.70. The interaction between block and players is significant, *F*(3, 401) = 832.02, *p* < 0.001, *η_p_^2^* = 0.68. The results of simple effect analysis showed that, for player 2, the amount of investment in block 2 (8.07) was significantly higher than that in block 1 (1.99), and the amount of investment in block 2 (1.85) was significantly lower than that in block 1 (8.40) in player 3, indicating that participants pay more attention to the ongoing behavior of their partner than their past behavior. The interaction between player and gender was significant, *F*(3, 401) = 11.63, *p* < 0.001, *η_p_^2^* = 0.03. The results of simple effect analysis showed that for player 1 and player 2, the investment amount by male participants (8.43, 5.22) was significantly higher than that invested by female participants (8.02, 4.85), while for player 4, the investment amount by female participants (2.31) was significantly higher than that invested by male participants (1.59). The interaction between block, player and gender was significant, *F*(3, 401) = 5.80, *p* < 0.01, *η_p_^2^* = 0.014. The results of simple effect analysis showed that, in block 1, the investment amount by male participants (8.63) was significantly higher than that invested by female participants (8.16) for player 3. For player 4, the investment by female participants (2.46) was significantly higher than invested by male participants (1.78). In block 2, for player 1 and player 2, the investment amount by male participants (8.91, 8.58) was significantly higher than that invested by female participants (8.04, 7.57), and for player 4, investment by female participants (2.16) was significantly higher than invested by male participants (1.40). See Table 4 for details.

**Table 4:** The amount of investment under different conditions in FF versions (*M* ± *SD*)


GENDER	BLOCK	PLAYER 1(TR)	PLAYER 2(UN*)	PLAYER 3(TR*)	PLAYER 4(UN)

male	1	8.37 ± 2.41	1.87 ± 2.76	8.64 ± 2.09	1.78 ± 2.46

	2	8.91 ± 1.95	8.58 ± 2.37	1.71 ± 2.68	1.40 ± 2.63

female	1	8.00 ± 2.48	2.12 ± 2.21	8.16 ± 2.16	2.45 ± 2.36

	2	8.04 ± 2.66	7.56 ± 2.82	1.98 ± 2.78	2.16 ± 3.14


*Notes:* TR = trustworthy, UN = untrustworthy; * = inconsistent.

These results revealed that individuals invested more in trustworthy players than in untrustworthy players; when making trust decisions, individuals were influenced by the player’s face emotion, but when the face emotion is inconsistent with the behavior pattern (such as untrustworthy players with happy facial emotion), individuals were more inclined to make decisions based on the behavior pattern.

## Experiment 2 The effect of high-low arousal of happy-angry facial emotion and trustworthy behavior on decision-making involving trust

### Methods

#### Participants

We conducted a power analysis using G*power following prior studies (Campellone & Kring, 2013; Faul et al., 2007), with the result showing that 1–β = 0.99 (effect size = 0.25, alpha = 0.05, total sample size = 116, number of groups = 4, number of measurements = 4, corr among rep measures = 0.5, nonsphericity correction ɛ = 1). One hundred sixteen undergraduates (*M*_age_ = 19.23, *SD*_age_ = 1.75, 59 males) from Shandong Normal University participated in the present study and received gifts for their participation in the experiment.

All human participating procedures used in the present study were conducted in the light of the ethical standards of the academic committee of Shandong Normal University and certify that the study was performed in accordance with the ethical standards as laid down in the 1964 Declaration of Helsinki and its later amendments or comparable ethical standards. Participants written the informed consent and were informed that they can terminate the experiment at any time.

#### Experimental Design

A 2 (facial emotion: happy vs angry) × 2 (arousal: high vs low) × 2 (partner’s behavior: trustworthy vs untrustworthy) × 2 (version: behavior first vs facial emotion first) mixed design was adopted in the current study with version and arousal as between-subject variables and facial emotion and partner’s behavior as within-subject variables.

The pictures in the current study were selected from the Chinese emotional picture system revised by Bai et al. (2005). A total of eight high- and low-arousal happy and angry facial emotion pictures were selected, half were men and half were women, which were consistent in attractiveness (two with high-arousal happy facial emotion; arousal was 6.00 and 6.67, respectively, attractiveness was 6.43 and 5.51, respectively. Two with low-arousal happy facial emotion; arousal was 3.67 and 3.00, respectively, attractiveness was 5.61 and 6.23, respectively. Two with high-arousal angry facial emotion; arousal was 7.33 and 8.33, respectively, attractiveness was 4.11 and 4.50, respectively. Two with low-arousal angry facial emotion; arousal was 3.33 and 4.67, respectively, attractiveness was 4.00 and 3.52, respectively. See Appendix B).

The dependent variable was the amount of the participant’s investment. Experimental setup is same as experiment 1. See more details in Tables 5 and 6.

**Table 5:** Facial emotion and trustworthiness of four players in two version.


PLAYER	BF VERSION		FF VERSION	
	
	BLOCK 1	BLOCK 2	BLOCK 1	BLOCK 2

Player 1	TR	TR + HAP + L	TR + HAP + L	TR

Player 2	UN	UN + HAP* + L	UN + ANG + L	TR*

Player 3	TR	TR + ANG* + L	TR + HAP + L	UN*

Player 4	UN	UN + ANG + L	UN + ANG + L	UN


*Notes:* TR = trustworthy, UN = untrustworthy; HAP = happy facial emotion; ANG = angry facial emotion; H = high arousal, L = low arousal; * = inconsistent.

**Table 6:** Facial emotion and trustworthiness of four players in two version.


PLAYER	BF VERSION		FF VERSION	
	
	BLOCK 1	BLOCK 2	BLOCK 1	BLOCK 2

Player 1	TR	TR + HAP + H	TR + HAP + H	TR

Player 2	UN	UN + HAP* + H	UN + ANG + H	TR*

Player 3	TR	TR + ANG* + H	TR + HAP + H	UN*

Player 4	UN	UN + ANG + H	UN + ANG + H	UN


*Notes:* TR = trustworthy, UN = untrustworthy; HAP = happy facial emotion; ANG = angry facial emotion; H = high arousal, L = low arousal; * = inconsistent.

#### Procedure

The procedure was the same as in experiment 1, except that there were two programs in the experiment that included the different version and high-low arousal.

#### Data Analysis

Statistical analyzes were performed using SPSS 16.0.

### Results

According to the first hypothesis, participants will invest significantly more in trustworthy players than in untrustworthy players. The current study analyzed the first block of the BF version, and the results showed that the amount invested in trustworthy players (7.23 ± 2.69) was significantly greater than that in untrustworthy players (3.28 ± 3.05), *t* (1862) = 37.85, *p* < 0.001, *d* = 1.37.

To test the second hypothesis, the present study analyzed the first block of the FF version and BF version via repeated ANOVA of the 4 (player) × 2 (version) × 2 (gender) design. The results showed that the main effect of player was significant, *F*(3, 1810) = 1756.46, *p* < 0.001, *η_p_^2^* = 0.493, the main effect of gender was significant, *F*(1, 1810) = 18.56, *p* < 0.001, *η_p_^2^* = 0.01, and the main effect of version was significant, *F*(1, 1810) = 12.31, *p* < 0.001, *η_p_^2^* = 0.007. The interaction between player and version was significant, *F*(3, 1810) = 32.79, *p* < 0.001, *η_p_^2^* = 0.018. Further simple effect analysis showed that for player 1 and player 3, the amount of investment in the FF version (7.66, 7.60) was significantly higher than that in the BF version (7.23, 7.25); for player 2 and player 4, the amount of investment in the FF version (2.29, 2.66) was significantly lower than that in the BF version (3.20, 3.45). The interaction between player and gender was significant, *F*(3,1810) = 27.63, *p* < 0.001, *η_p_^2^* = 0.015. Further simple effect analysis showed that for player 1, the investment amount by male participants (7.33) was less than that invested by female participants (7.57), while for player 2 and player 4, the investment amount by male participants (2.99, 3.43) was significantly higher than that invested by female participants (2.50, 2.68). The interaction between gender and version was significant, *F*(3, 1810) = 45.51, *p* < 0.001, *η_p_^2^* = 0.025. Further simple effect analysis showed that in the BF version, the investment amount by male participants (5.65) was significantly higher than that invested by female participants (4.97), and there was no significant difference in the FF version. The interaction of player, gender and version was significant, *F*(3, 1810) = 3.10, *p* < 0.05, *η_p_^2^* = 0.002. Further simple effect analysis showed that, in the BF version with four players, the amount of investment by female participants (7.20, 2.85, 6.88, 2.92) was lower than that invested by male participants (7.45, 3.55, 7.63, 3.99). However, in the FF version, for players 1 and 3, female participants (8.12, 7.82) placed more trust than male participants (7.21, 7.37), for player 4, male participants (2.88) invest more than female participants (2.43). See Table 7 for details.

**Table 7:** The amount of investment under different conditions in the two versions of block1 (*M* ± *SD*).


GENDER	VERSION	PLAYER 1(TR)	PLAYER 2(UN)	PLAYER 3(TR)	PLAYER 4(UN)

male	Behavior	7.45 ± 2.82	3.55 ± 3.26	7.62 ± 2.80	3.98 ± 3.25

	Facial emotion	7.21 ± 2.78	2.43 ± 2.50	7.37 ± 2.70	2.88 ± 2.62

female	Behavior	7.02 ± 2.54	2.84 ± 2.75	6.88 ± 2.54	2.92 ± 2.83

	Facial emotion	8.12 ± 2.39	2.15 ± 2.16	7.82 ± 2.44	2.43 ± 2.51


*Notes:* TR = trustworthy, UN = untrustworthy.

To test the third hypothesis, we separately analyzed the BF version and FF version. First, we carried out a repeated ANOVA of the 4 (player) × 2 (block) × 2 (gender) design for the BF version. The results showed that the main effect of player was significant, *F*(3, 925) = 1699.55, *p* < 0.001, *η_p_^2^* = 0.648, and the main effect of block was significant, *F*(1, 925) = 39.09, *p* < 0.001, *η_p_^2^* = 0.041. The main effect of gender was significant, *F*(1, 925) = 87.23, *p* < 0.001, *η_p_^2^* = 0.086. The interaction between block and player was significant, *F*(3, 925) = 167.81, *p* < 0.001, *η_p_^2^* = 0.154. Further simple effect analysis showed that the amount of investment in player 1 (7.23, 8.27 and player 3 (7.24, 7.80) was significantly larger than that in player 2 (3.21, 1.83) and player 4 (3.39, 1.76) in both block 1 and block 2. See Table 8 for details.

**Table 8:** The amount of investment under different conditions in BF versions (*M* ± *SD*).


GENDER	BLOCK	PLAYER 1(TR)	PLAYER 2(UN*)	PLAYER 3(TR*)	PLAYER 4(UN)

male	1	7.42 ± 2.81	3.55 ± 3.25	7.60 ± 2.79	3.89 ± 3.26

	2	8.6 ± 2.43	2.22 ± 3.03	8.00 ± 2.92	2.06 ± 2.71

female	1	7.04 ± 2.53	2.88 ± 2.75	6.87 ± 2.57	2.89 ± 2.84

	2	7.95 ± 2.37	1.45 ± 1.98	7.61 ± 2.54	1.46 ± 2.07


*Notes:* TR = trustworthy, UN = untrustworthy; * = inconsistent.

In addition, we also performed a repeated ANOVA of the 4 (player) × 2 (block) × 2 (gender) design for the FF version. The results showed that the main effect of player was significant, *F*(3, 852) = 1125.42, *p* < 0.001, *η_p_^2^* = 0.569. The main effect of block was significant, *F*(1, 852) = 6.68, *p* < 0.05, *η_p_^2^* = 0.008. The interaction between block and players was significant, *F*(3, 852) = 1034.42, *p* < 0.001, *η_p_^2^* = 0.549. The results of simple effect analysis showed that, for player 2, the amount of investment in block 2 (7.34) was significantly higher than that in block 1 (2.29), and for player 3, the amount of investment in block 2 (2.34) was significantly lower than that in block 1 (7.61), indicating that participants paid more attention to the updated behavior of partners than their past behavior. The interaction between player and gender was significant, *F*(3, 852) = 5.82, *p* < 0.01, *η_p_^2^* = 0.007. The results of simple effect analysis showed that for player 2, the investment amount by male participants (4.98) was significantly higher than that invested by female participants (4.66), while for player 3, the investment amount by female participants (5.14) was significantly higher than that invested by male participants (4.80). The interaction of block and gender was significant, *F*(3, 852) = 5.82, *p* < 0.01, *η_p_^2^* = 0.007. The results of simple effect analysis showed that the investment amount by male participants (4.93) was significantly lower than that invested by female participants (5.15) in block 1. The interaction between block, player and gender was significant, *F*(3, 852) = 10.85, *p* < 0.001, *η_p_^2^* = 0.013. The results of simple effect analysis showed that in block 1, the investment amount by female participants (7.32, 7.89) was significantly higher than that invested by male participants (7.16, 8.1) for players 1 and 3. For player 4, the investment by female participants (2.37) was significantly lower than that invested by male participants (2.87). In block 2, for player 1, the investment amount by male participants (8.09) was significantly higher than that invested by female participants (7.58). See Table 9 for details.

**Table 9:** The amount of investment under different conditions in FF versions (*M* ± *SD*).


GENDER	BLOCK	PLAYER 1(TR)	PLAYER 2(UN*)	PLAYER 3(TR*)	PLAYER 4(UN)

male	1	7.16 ± 2.82	2.42 ± 2.50	7.32 ± 2.69	2.81 ± 2.66

	2	8.09 ± 2.85	7.54 ± 3.24	2.27 ± 3.01	1.99 ± 2.77

female	1	8.17 ± 2.37	2.17 ± 2.19	7.89 ± 2.41	2.37 ± 2.51

	2	7.58 ± 3.21	7.14 ± 3.35	2.39 ± 2.69	2.11 ± 2.66


*Notes:* TR = trustworthy, UN = untrustworthy; * = inconsistent.

Finally, we tested hypothesis 4, according to which individuals with happy facial emotion with high arousal were more likely to be trusted than individuals with happy facial emotion with low arousal, and individuals with angry facial emotion with high arousal were more likely to be distrusted than individuals with angry facial emotion with low arousal. We analyzed block 1 of the FF version with high arousal and low arousal and performed a repeated measurement ANOVA of the 4 (player) × 2 (arousal) × 2 (gender) design. The results showed that the main effect of player was significant, *F*(3, 874) = 1286.92, *p* < 0.001, *η_p_^2^* = 0.596. The interaction between player and arousal was significant, *F*(3, 874) = 10.84, *p* < 0.001, *η_p_^2^* = 0.012. For players 1 and 3, the amount of investment in players with high arousal (7.96, 7.78) was significantly higher than that in players with low arousal (7.37, 7.42). For players 2 and 4, the amount of investment in players with high arousal (2.05, 2.44) was significantly lower than that in players with low arousal (2.53, 2.88). The interaction between player and gender was significant, *F*(3, 874) = 15.21, *p* < 0.001, *η_p_^2^* = 0.017. The results of simple effect analysis showed that for players 1 and 3, the investment amount by female participants (8.13, 7.83) was significantly higher than that invested by male participants (7.20, 7.37). For players 2 and 4, the investment amount by female participants (2.14, 2.44) was significantly lower than that invested by male participants (2.43, 2.89). The interaction among player, arousal and gender was significant, *F*(3, 874) = 6.87, *p* < 0.001, *η_p_^2^* = 0.008. The results of simple effect analysis showed that in high arousal, for players 1, 2, 3 and 4, gender was not significant. In the low arousal, for players 1 and 3, the investment amount by female participants (8.10, 7.69) was significantly higher than that invested by male participants (6.63, 7.14). For players 2 and 4, the investment amount by female participants (2.26, 2.42) was significantly lower than that invested by male participants (2.80, 3.33). See Table 10 for details.

**Table 10:** The amount of investment under different arousal (*M* ± *SD*).


GENDER	AROUSAL	PLAYER 1(TR)	PLAYER 2(UN)	PLAYER 3(TR)	PLAYER 4(UN)

male	high	7.76 ± 2.72	2.07 ± 2.10	7.59 ± 2.96	2.44 ± 2.47

	low	6.63 ± 2.72	2.79 ± 2.81	7.14 ± 2.39	3.33 ± 2.70

female	high	8.15 ± 2.45	2.02 ± 2.01	7.97 ± 2.46	2.45 ± 2.50

	low	8.10 ± 2.34	2.26 ± 2.28	7.69 ± 2.42	2.42 ± 2.53


*Notes:* TR = trustworthy, UN = untrustworthy.

These results revealed that individuals invested more in trustworthy players than in untrustworthy players; when making trust decisions, individuals were influenced by the player’s face emotion, but when the face emotion is inconsistent with the behavior pattern (such as untrustworthy players with happy facial emotion), individuals were more inclined to make decisions based on the behavior pattern; individuals invested more money to players with high-arousal happy facial emotion than those with low-arousal happy facial emotion, and individuals invested less money to players with high-arousal angry facial emotion than those with low-arousal angry facial emotion.

## General Discussion

The present study uses the trust game to verify the influence of happy and angry facial emotions and trustworthy behavior on trust decision-making and discusses the influence of facial emotional arousal on individual trust decision-making. In experiment 1, we used the repeated interactive version of the trust game to explore the influence of the facial emotion of happiness and anger and trustworthy behavior on individual trust decision-making in the context of Chinese culture. Experiment 2 further explored the influence of facial emotional arousal on individual trust decision-making.

The first question discussed in the present study is whether individuals can accurately identify whether players can be trustworthy in a repeated interaction with players. The results of the two experiments show that individuals can learn the behavior patterns of players in the repeated interaction with four players, and the subjects invest more in trustworthy players than untrustworthy players. This confirms the results obtained from previous studies (Fu et al., 2018) within the context of Chinese culture. In the block with only trustworthy behavior information, the individual can accurately identify the player’s pattern, speculate on the player’s follow-up behavior, and make judgements and decisions accordingly. As the social norms theory holds, trustworthy behavior of the individual follows the principle of reciprocity, that is, the individual will reward the well-intentioned behavior and punish the hostile behavior (Evans & Krueger, 2009; Falk & Fischbacher, 2006; Gouldner, 1960; Kruegeret al., 2008).

The second problem solved in this study is the role of facial emotion in trust decision-making. The results support the hypothesis that happy and angry facial emotions will provide corresponding information for judgement and decision-making in the repeated interaction with the player. This finding shows that the participants are affected by facial emotion in trust decision-making. The findings of the present study confirm the results of previous study exploring the effect of facial features on the individual initial investment amount in the context of Chinese culture (Chang et al., 2010; van’t Wout & Sanfey, 2008). The result of this experiment validates the “feeling as information model”. Emotion can be used as information to directly affect the individual’s judgement and decision-making process. Positive emotions influence individuals to make positive decisions, while negative emotions influence individuals to make negative decisions (Schwarz & Clore,1988). In addition, according to the emotion as a social information model, just as emotion provides information for decision-making (Schwarz & Clore,1983), facial emotion also provides information for the observer, which may affect the individual’s behavior (Kleef & Gerben, 2009). In the present study, happy facial emotion indicated the trustworthiness of the players, while angry facial emotion indicated the untrustworthiness of the players. The model also stipulates that when individuals observe other people’s facial emotions, they will make a series of inferences based on facial emotions. Therefore, people placed more investment amount in players with happy facial emotion and less in those with angry facial emotion.

The third problem solved in the current study is what information is important in individual trust decision-making when facial emotional information and trustworthy behavioral information is inconsistent. The results of two experiments showed that participants invested less in players who were happy but untrustworthy, and more in those who were angry but trustworthy. This is consistent with the finding of previous studies (Campellone & Kring, 2013), suggesting that facial emotions of happiness and anger do not affect established patterns of behavior. In the case when the behavior pattern of a player has been formed and there is conflict between facial emotion and behavior information, the individual will only make decisions according to the established behavior pattern of the player. Only when the individual has not yet learned the player’s behavior pattern does facial emotion affect the subjects’ choice. This is consistent with the finding that the best predictor of an individual’s extent of trustworthiness is their behavior in a previous interaction with (Axelrod & Hamilton, 1981; King-Casas et al., 2005).

In addition, we also explored whether people make judgements and decisions according to the players’ current behavior when the players’ behavior is inconsistent between past and present. The results of two experiments showed that when the player’s behavior changes, the amount of investment will also change with the player’s ongoing behavior, that is, the player’s ongoing behavior affects the subject’s judgement and decision-making. This is consistent with the results of previous studies (Chang et al., 2010; King-Casas et al., 2005). These results support the following two points: first, when the behavior of the social partner changes, the individual’s decision-making will also change according to the partner’s ongoing behavior; second, when the facial emotion and behavior information of the interacting partner conflict, if the behavior pattern remains unchanged, the role of facial emotion is greatly reduced, and there is more dependency on behavioral information. Researchers believe that individuals make social judgements and decisions based on their first impressions of each other at first, but with repeated interactions, individuals’ judgements and decisions will be constantly updated (Chang et al., 2010) with the information they obtain about partners’ behavior.

In the fourth question of the present study, we explore the influence of emotional arousal on the individual trust decision-making process. We hypothesized that players with high-arousal happy facial emotion will receive more investment than those with low arousal, while players with high-arousal angry facial emotion will receive less investment. The results confirmed this hypothesis. Therefore, we think that facial emotional arousal also plays an important role in individual trust decision-making. According to the dimension theory of emotion (Russell, 2003), arousal, as the other dimension of emotion, will also have an impact on decision-making. The results of this study confirm that facial emotional arousal affects the amount of trust investment. It enriches the dimensional theory of emotion.

Finally, we discuss the fifth question, which addresses gender differences in trust decision-making. The results showed that, in the context of Chinese culture, female subjects generally invested less than male subjects, which confirms the findings of previous studies on gender differences in trust games (Campellone & Kring, 2013; Van Den Akker et al., 2020). Existing research shows that female participants are more inclined to risk-aversion in decision-making involving risk (Byrnes et al., 1999; Croson & Gneezy, 2009). Moreover, trust decisions often endure considerable risk and uncertainty (Chang et al., 2010). Although in the trust game, the amount of individual investment is reciprocated by the partners, but the decisions of individual investing are still risky in the interaction with the partners (Zhang et al., 2016).

Based on the above findings, we believe that trust decision-making is dynamic. In the repeated interaction, our results showed that regardless of the player’s facial emotion and that of arousal, the individual only invested more money in the player who showed trustworthy behavior. Player’s facial emotion can provide some information for the individual’s judgement and decision-making process, but this situation only occurs under the condition when the player’s facial emotion is consistent with the behavior pattern. Taken together, our findings have some implication for social interactions and trust decision-making. Based on previous studies in the West, we selected facial emotion of Chinese people to verify the existing findings in the context of the Chinese culture and obtained similar results. Therefore, we preliminarily speculate that the choices in trust decision-making are similar in the context of Eastern and Western cultures. In addition, on the basis of previous studies, the present study further explored the impact of emotional arousal on trust decision-making and found an important result, that is, players with high-arousal facial emotion tend to invoke extreme trust decision-making. Therefore, the present study also expands our knowledge of the influence of facial emotion on trust decision-making.

### Limitations and Future Direction

In the past, most of the studies on the influence of facial emotion and trustworthy behavior on trust decision-making were carried out in the context of Western culture (Campellone & Kring, 2013; Chang et al. 2010). In this study, in the background of Chinese culture, the facial emotion images of Chinese individuals were selected as stimulating materials, and Chinese subjects were selected to explore the influence of facial emotion and trustworthy behavior on trust decision-making. The present study was only conducted in Chinese culture; it did not explore the impact of subjects with different cultural backgrounds on facial emotional processing. Therefore, future studies can explore whether there are differences in the effects of facial emotion and trust behavior on trust decision-making in different cultural contexts.

Secondly, in terms of facial emotion selected, the current study discussed the influence of happy and angry facial emotions on individual trust decision-making. Future studies can select more facial emotions to explore the different impacts on trust decision-making. In addition, the present study only explored the influence of facial emotion and trust behavior on trust decision-making from the behavioral level and did not explore the neural mechanism of facial emotion and trustworthy behavior affecting trust decision-making. Previous studies have explored the role of medial prefrontal cortex in individual trust decision-making (Fouragnanetal, 2013; Krueger et al., 2008; McCabe et al., 2001; Moretto, Sellitto, & di Pellegrino, 2013). The caudate nucleus can learn the trustworthiness of an interaction from indirect interaction experience (King-Casas et al., 2005; Wardle et al., 2013), the relationship between the amygdala and the trust game (Adolphsetal, 2005; Koscik & Tranel, 2011; Todorov, 2012; van Honk et al., 2013), and the relationship between the anterior insula and uncertainty and risk perception (Riedl & Javor, 2012). The above studies only focused on the brain activity of the trust game and did not explore the role of facial emotion in trust decision-making. Therefore, future studies should explore the brain areas where facial emotion affects trust decision-making.

Thirdly, regarding the dynamic changes in trust decision-making, this study solely examines how individuals’ behaviors adapt to variations in player behavior patterns and facial expressions. However, it fails to provide a detailed investigation into the process of behavioral changes, including when these changes occur during interactions. Therefore, future studies could explore the dynamics of trust decision-making in detail, which will be more conducive to observing the dynamic changes in individuals’ trust decision-making.

Finally, the subjects selected in this study are college students. Considering the extensibility of the experimental results, future studies can explore how different groups of subjects are affected by facial emotions when making trust decisions.

## Conclusion

In the repeated interaction with four players, individuals can learn the behavior patterns of players, and people invest more in trustworthy players than untrustworthy players. Individuals invest more in trustworthy players with happy facial emotions than those no facial emotion, while untrustworthy players with angry facial emotions receive less investment than those with no facial emotion. The results of both happy and angry facial emotions verify that the high-arousal facial emotion will result in a more extreme individual investment. When the players’ facial emotion and behavior are inconsistent, the individual will judge according to the player’s current behavior, rather than the facial emotion or past behavior.

## Data Accessibility Statements

The data that support the findings of this study are available from the corresponding author upon reasonable request.

## Additional File

The additional file for this article can be found as follows:

10.5334/pb.1214.s1 Appendixes. Appendix A to B.
