# Supplementary material for: Trust or Distrust: The Effect of Facial Emotion and Trustworthy Behavior on Trust Decision-Making
Source: Psychol Belg. 2023 Aug 17;63(1):105–19. doi: 10.5334/pb.1214 (PMC10437140; doi:10.5334/pb.1214)
Supplement: Appendixes. — Appendix A to B. [file pb-63-1-1214-s1.pdf]

Appendix A

Experiment 1

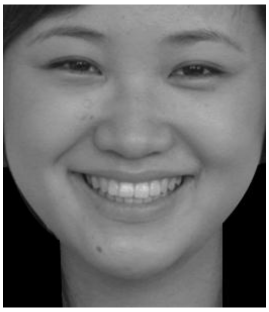

Fig. A.1

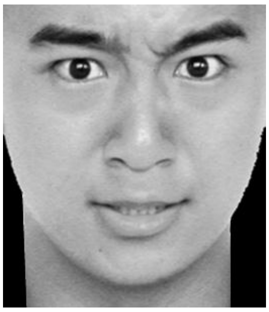

Fig. A.2

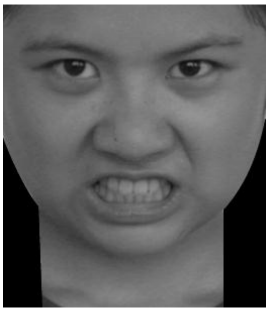

Fig. A.3

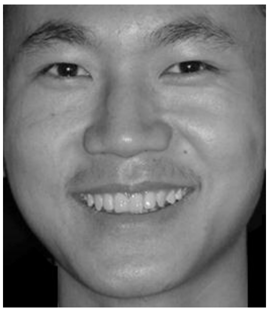

Fig. A.4

Appendix B

Experiment 2：Low Arousal

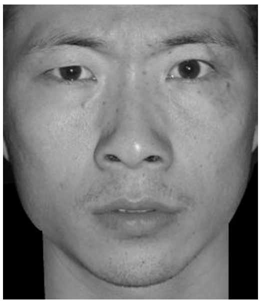

Fig. B.1

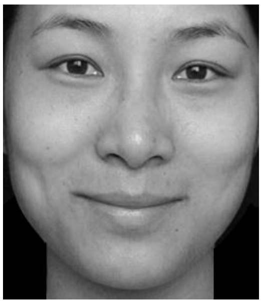

Fig. B.2

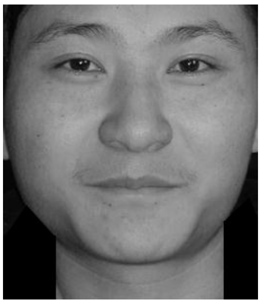

Fig. B.3

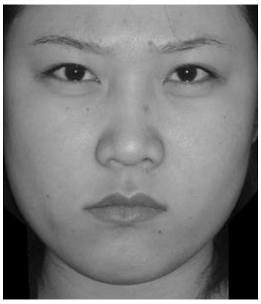

Fig. B.4

High Arousal

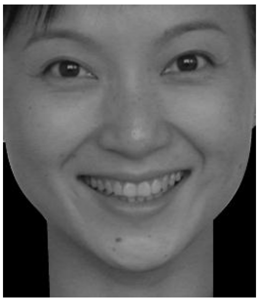

Fig. B.5

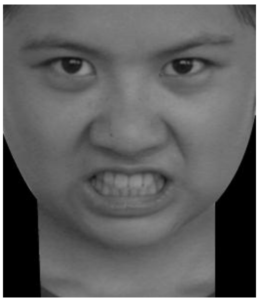

Fig. B.6

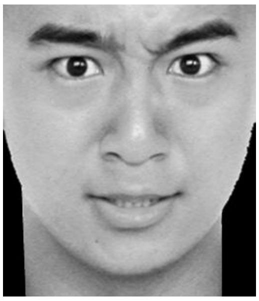

Fig. B.7

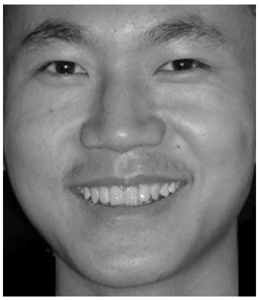

Fig. B.8
